# Endoscopic versus Open Bursectomy for Prepatellar and Olecranon Bursitis

**DOI:** 10.7759/cureus.2374

**Published:** 2018-03-27

**Authors:** Gokhan Meric, Serdar Sargin, Aziz Atik, Aydin Budeyri, Ali Engin Ulusal

**Affiliations:** 1 Department of Orthopaedics and Traumatology, Yeditepe University, Faculty of Medicine, Istanbul, Turkey; 2 Department of Orthopaedics and Traumatology, Balikesir University, Faculty of Medicine, Balikesir, Turkey; 3 Department of Orthopaedics and Traumatology, SANKO University, Faculty of Medicine, Gaziantep, Turkey

**Keywords:** endoscopic, bursitis, olecranon bursitis, prepatellar bursitis, minimally invasive surgery, endoscopic bursectomy, open bursectomy, endoscopic bursectomy, elbow arthroscopy, knee arthroscopy

## Abstract

Objectives

Bursitis of the olecranon and the patella are not rare disorders, and conservative management is successful in most cases. However, when patients do not respond to conservative treatment, open excisional surgery or, recently, endoscopic bursectomy, can be used. The aim of this study was to evaluate the results of open and endoscopic treatments of olecranon and prepatellar bursitis.

Patients and methods

Forty-nine patients (37 male and 12 female), who were treated with endoscopic bursectomy (25 patients) or open bursectomy (24 patients) were included in this study. Thirty patients had olecranon bursitis, while 19 patients had prepatellar bursitis. The patients’ average age was 61.1 ± 12.3 (range 33-81) years. All of the patients’ hospitalization and surgery times were recorded.

The satisfaction of the patients was evaluated with a satisfaction scoring system, as well as by evaluating residual pain, the range of joint movement, and the cosmetic results of the procedure.

Results

The average follow-up time was 16 ± 9 months (range 12–27). The median operation time was 23.2 ± 3.5 minutes for the endoscopic bursectomy group and 26.4 ± 6.8 minutes for the open bursectomy group. The median hospitalization time was 0.56 ± 0.5 days (range 0-1 day) for the endoscopic group and 1 ± 0 days for the open bursectomy group (P<0.01).

According to the patient satisfaction questionnaire, the endoscopic bursectomy group’s score was 8.5 ± 1.3 (range 5-10), and the open bursectomy group’s score was 5.29 ± 1.8 (range 1-9) (P<0.01).

Conclusion

Endoscopic bursectomy is a time-saving and efficient surgical treatment option for patients with prepatellar and olecranon bursitis.

## Introduction

Olecranon and prepatellar bursitis are common conditions. They are caused by repetitive minor trauma, acute trauma, and inflammatory diseases such as gout and rheumatoid arthritis [1-4]. The patients usually complain about pain and swelling.

Most of the aseptic bursitis cases responded to conservative treatment such as ice, rest, anti-inflammatory drugs, aspiration, and local corticosteroid and lidocaine injections. When patients do not respond to conservative treatment, surgical treatment may be required [5-6].  Surgical treatment options are aspiration and irrigation, incision and drainage, and total excision through an open or endoscopic total excision [1,7-8].

Endoscopic bursectomy has been introduced for the surgical treatment of bursitis [6]. Endoscopic bursal resection was first described in 1990 by Kerr and Carpenter [6]. Kerr reported that the arthroscopic resection of prepatellar and olecranon bursae was a practical and useful operation, and that this technique can prevent wound complications. There are also several studies in the literature that report that endoscopic bursectomies have a short recovery time, reduce costs, save time, and shorten hospital stays [6, 9-11]. However, almost all of these studies were case studies. Also, open bursectomies can cause skin complications. In the literature, Ogilvie-Harris and Gilbart treated 50 patients with prepatellar and olecranon bursitis using endoscopic bursectomy and there were no skin complications or recurrences [11]. The purpose of this study was to compare open and endoscopic techniques for the treatment of olecranon and prepatellar bursitis.

## Materials and methods

Our study included endoscopic and open bursectomies performed for olecranon and prepatellar bursitis between September 2010 and November 2012 at Balikesir University, Faculty of Medicine, Department of Orthopaedics and Traumatology, Balikesir, Turkey. A total of 49 patients (37 male and 12 female) with prepatellar and olecranon bursitis were included in this study. Nineteen patients had prepatellar bursitis, and 30 patients had olecranon bursitis. The study was carried out according to the Declaration of Helsinki (DoH), and an institutional review board gave approval for it.

Twenty-four patients underwent excisional bursectomy, and 25 patients underwent endoscopic bursectomy. No significant differences were found among endoscopic and open groups in terms of age, sex, etc.

Diagnoses were made based on clinical and radiological investigation. All the patients initially received conservative treatment and aspiration, or a steroid injection into the olecranon or prepatellar bursal cavity. Patients who had not responded to conservative treatment for three months were included in the study. Patients with inflammatory diseases and bony abnormalities were excluded from the study. Preoperatively, endoscopic bursectomy patients were given local steroid injections (40 mg methylprednisolone) for an average of 1.6 ± 0.7 times (range 1–4) and underwent aspiration for an average of 2.8 ± 0.7 times (range 0–3). Open bursectomy patients received local steroid injections at an average of 1 ± 0.7 times (range 0-2) and underwent aspiration at an average of 1.2 ± 0.8 times (range 0–3).

Open bursectomy technique

**Figure 1:**
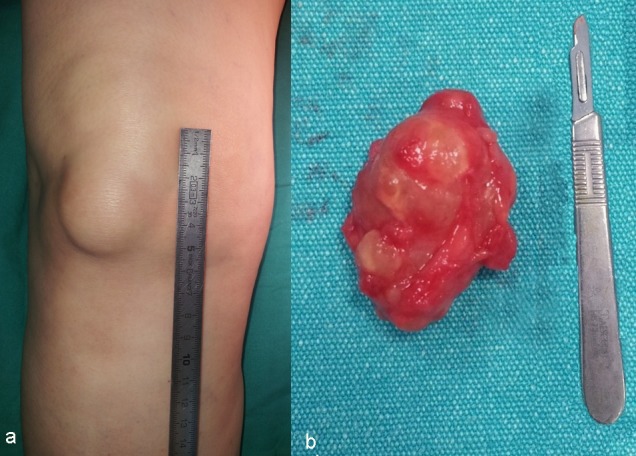
a) Clinical view of a right prepatellar bursitis; b) open excised prepatellar bursa

Open bursectomy was performed with the patient in a supine position, with tourniquet control under regional anesthesia. For olecranon bursitis, a longitudinal radial incision, beginning from the lateral border of the triceps muscle to the distal border of the bursitis, was used to avoid the ulnar nerve. For prepatellar bursitis, a median longitudinal incision was used. The entire bursa was removed till the subcutaneous fatty tissue appeared (Figure 1). Then, the potential empty space was closed with subcutaneous 4/0 absorbable sutures to avoid hematoma. The skin was closed with 3/0 nylon sutures. Any drains left in the place were removed next day.

Endoscopic bursectomy technique

Endoscopic bursectomy was performed with the patient in a supine position with tourniquet control. The skin was prepared in an aseptic manner. Endoscopic bursectomy was performed under local anesthesia. A 10 ccs 2% lidocaine solution was injected through the two portal (medial and lateral) sites, and then into the cavity. Regional anesthesia was performed for patients who felt pain.

**Figure 2:**
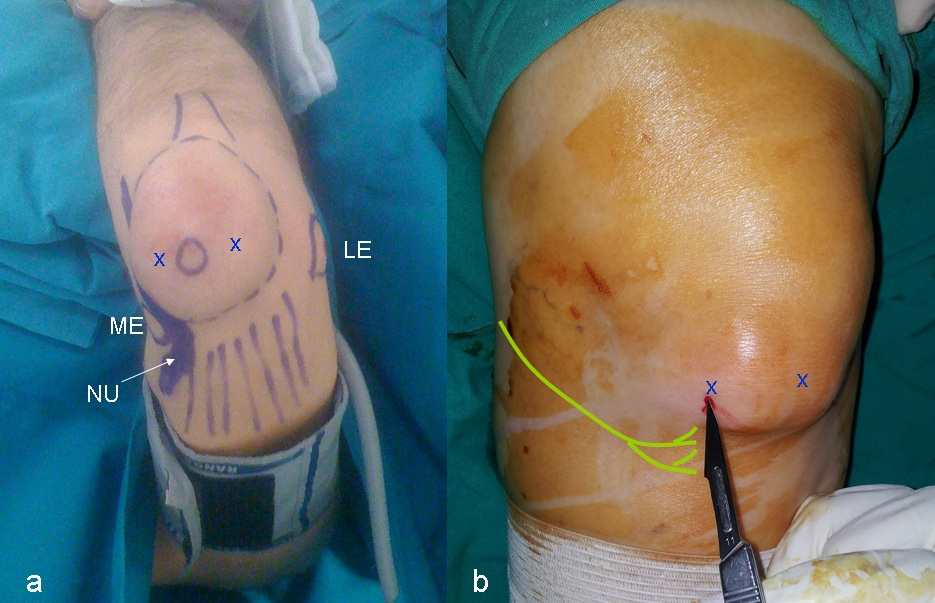
a) Clinical view of the patient with left olecranon bursitis and zones that need attention (ME: medial epicondyle, LE: lateral epicondyle, NU: nerveus ulnaris); b) minimal prepatellar portals opened with no. 11 scalpel (yellow drawing: infrapatellar branch of saphenous nerve; X marks: the portal for endoscopy)

**Figure 3:**
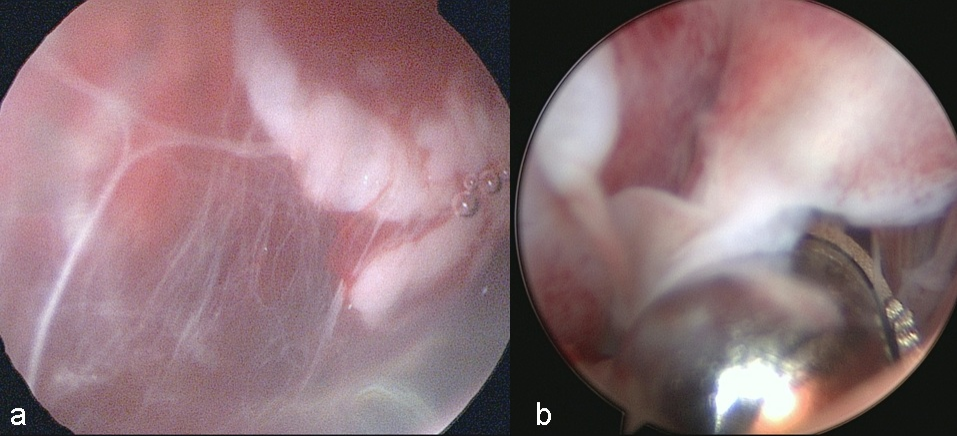
a) Intraoperative view of endoscopic bursectomy in left knee with prepatellar bursitis; anteromedial portal was opened a little bit laterally to avoid any damage to infrapatellar nerve; b) endoscopic view of the prepatellar bursitis

**Figure 4:**
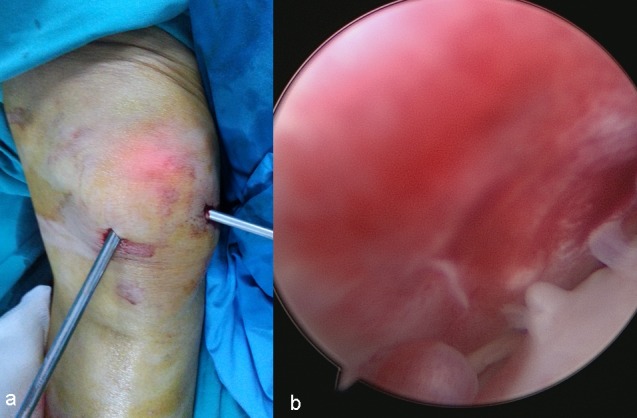
a) Endoscopic view of the hypervascularised synovium; b) bursal wall was debrided with shaver

For olecranon bursitis, the medial portal was opened anteriorly to protect the ulnar nerve (Figure 2), and for prepatellar bursitis, the anteromedial portal was opened laterally to protect the infrapatellar nerve (Figure 3). A 30-degree endoscope was used for monitoring and a 4.5-mm shaver was used for synovectomy. The endoscope and shaver were directed into the bursal sac. The bursal cavity was first visualized endoscopically. All bursal tissue was resected while taking care not to damage the skin (Figure 4). The tip of the shaver was always directed toward the joint surface to avoid any injury to the subcutaneous tissue or neurovascular structures. The portals were closed with primary suturation. If any drain was left in place, it was removed the next day.

Postoperative period

Non-steroidal anti-inflammatory drugs and prophylactic antibiotics were given for two days. Elastic bandages and ice packing were used. For olecranon bursitis, the patient’s arm was placed in a broad arm sling with the elbow at 90 degrees of flexion to ease compression over the cavity. Gentle active range of motion exercises were allowed after the first day of the procedure. Wound dressings were changed every day after the procedure and the sutures were removed two weeks after the surgery if applied, and then the patients were left to return to daily activities.

Outcome measurements and statistical analysis

To evaluate patient satisfaction, a scoring system was designed. The satisfaction of the patients with the treatment was measured by a questionnaire ranging from 10 (extremely satisfied) to 1 (extremely dissatisfied), at the latest follow-up. Pain and function in comparison to preoperative status were also evaluated and rated as better, same, or worse. Cosmetic results were rated, ranging from 10 (significantly improved) to 1 (significantly worse). Hospitalization and surgery times and complications were also recorded.

All statistical analysis was performed via SPSS 20.0 for Windows (IBM Corporation, Armonk, NY, US). Continuous variables were expressed as mean ± standard deviation. Mann-Whitney U testing was used to compare the two groups. A P-value under 0.01 was considered statistically significant.

## Results

The average follow-up was 16 ± 9 months (range 12–27). The median operation times were 23.2 ± 3.5 minutes for the endoscopic bursectomy group, and 26.4 ± 6.8 minutes for the open bursectomy group (P=0.091). All endoscopic bursectomies were performed under local anesthesia. The median hospitalization time was 0.56 ± 0.5 days (range 0-1 day) for the endoscopic group and 1 ± 0 days for the open bursectomy group. These differences were statistically significant (P <0.01) (Table 1).

**Table 1:** Statistical results

Patient	Open Bursectomy	Endoscopic Bursectomy	P value
Operation time	26.4 ± 6.8 minutes	23.2 ± 3.5 minutes	0.091
Hospital stay time	1 ± 0 days	0.56 ± 0.5 days	<0.01
Patient questionnaire	5.29 ± 1.8 (range 1-9)	8.5 ± 1.3 (range 5-10)	<0.01

When the means of treatment satisfaction were compared, the endoscopic bursectomy group yielded superior scores over the open surgery group at 8.5 ± 1.3 (range 5-10) versus 5.29 ± 1.8 (range 1-9), respectively (P <0.01). All patients returned to work and their daily activities. There were no limitations on movement in the knee and elbow joints in all the cases.

Two patients undergoing open bursectomies had wound infections which were resolved with antibiotics. One patient had a fistula at the third week after the open bursectomy. This patient later underwent a through fistulectomy. One open olecranon bursectomy patient had recurrent swelling, which was resolved after aspiration. One patient with prepatellar bursitis had recurrent effusions after the endoscopic bursectomy. After aspiration, no more recurrences was observed. 

## Discussion

To our knowledge, this study is the first one that compares the results of these two techniques. Endoscopically-treated patients tolerated local anesthesia and did not require general anesthesia. Hospitalization times were significantly greater for the open surgical procedures. Patient satisfaction scores were higher for the endoscopic bursectomy group.

Endoscopic bursal resection was first described in 1990 by Kerr and Carpenter [6]. In their report, two patients with traumatic bursitis had good results, but one patient with inflammatory bursitis secondary to CREST (calcinosis, Raynaud’s phenomenon, esophageal dysmotility, sclerodactyly, telangiectasia) syndrome had an unsatisfactory result. They suggested that patients with any inflammatory cause of bursitis might not be good candidates for arthroscopic excision [6]. Kerr reported that arthroscopic resection of prepatellar and olecranon bursae was a practical and useful operation, and that this technique can prevent wound complications [12].

However, open bursectomies can cause skin complications. In the literature, Ogilvie-Harris and Gilbart treated 50 patients with prepatellar and olecranon bursitis using endoscopic bursectomies and there were no skin complications or recurrences [11]. There was one superficial infection that was treated with topical antibiotics and there were two cases of delayed wound healing. Tu et al. reviewed extrabursal endoscopic bursectomy techniques for the treatment of olecranon bursitis to avoid skin perforation [13]. They concluded that this technique lowers the rate of skin perforation, and thus avoids sinus tract progression.

General or local anesthesia can be used for endoscopic bursectomy. Nussbaumer et al. treated 50% of 13 patients with olecranon and prepatellar bursitis using endoscopic bursectomy under general anesthesia [10]. Huang and Yeh reported the treatment of 60 patients with post-traumatic prepatellar bursitis with endoscopic bursectomy [9]. They performed bursectomy under local anesthesia and concluded that endoscopic bursectomy is an effective and time-saving procedure for post-traumatic bursitis after the failure of conservative treatment. In our study, endoscopically-treated patients tolerated local anesthesia and did not require general anesthesia. We believe that effective management of local anesthesia is sufficient for most endoscopic bursectomies.

There are also several investigators who have reported complications with endoscopic bursa excisions [6-8, 11] (Table 1). Prepatellar bursitis commonly presents with thickening of the overlying skin. To reach into the prepatellar bursa, standard anteromedial and anterolateral knee arthroscopic portals can be used, but two lateral portals can also be utilized to protect the infrapatellar nerve [14]. Tendon injury during arthroscopic excision can be avoided by pointing the shaver blade away from the tendon and only turning the shaver on when in direct visualization of the blade tip. Epstein et al. reported a patient who had a patellar tendon rupture two weeks after an endoscopic prepatellar bursa resection [4]. They suggested that applying appropriate surgical techniques and proper shaver use can prevent tendon damage.

The most common complication resulting from open surgical treatment is the wound-healing problem [11, 15]. Poor healing of the wound, local tenderness, and decreased sensation at the scar site has been observed [16]. Ouayle and Robinson suggested excision of only the posterior half of the bursa during the open prepatellar bursectomy to prevent damage to the overlying skin [15]. Persistent sinuses may be seen after open septic bursectomy [17]. Hypoesthesia can be secondary to infrapatellar nerve damage. Stewart et al. suggested lateral incision and excision of the bursa as a single structure to ensure complete removal of all bursal tissue, and they reported that scar formation, infection, and prolonged recovery can be seen with open prepatellar bursal resection [18]. Dissection of the bursa may not be easy because the thick anterior wall is often stuck together with the skin, which can be damaged easily. In the literature, skin complications were seen in up to 20% of open bursectomy cases [8, 11, 19]. In our study, two patients had wound infections and one patient had a fistula after an open bursectomy. Our patients who were treated with endoscopic bursectomy did not have any wound healing issues; however, one had a recurrent effusion which resolved with aspiration.

Our study has several limitations. We used a patient satisfaction scale, but this scoring system has not been specifically validated for bursitis. This scoring system is a simple method of evaluating patient outcomes and cosmetic results. The aim of our study was to compare endoscopic and open bursectomies. Certainly, performing this study with isolated groups like prepatellar or olecranon bursitis patients could give us more reliable results. The pathophysiology of these conditions has similar etiology. In the literature, many similar studies were performed with both olecranon and prepatellar bursitis in the same study [6-7,11-12]. Also, our study lacks data concerning the cost of the procedure and long-term outcomes (Table 2).

**Table 2:** Comparison of our results with those in the literature

Study	Procedure	Patient Count	Follow-up (months)	Etiology	Location	Complications
Quayle-Robinson (1976) [8]	Open bursectomy	8	48	Traumatic	Prepatellar	Two patients, palsy of infrapatellar branch of saphenous nerve
Degreef-Smet (2006) [2]	Open bursectomy	37	-	Traumatic and one patient had lupus erythematosus	Olecranon	10 patients (27%) had wound-healing problems Eight patients (22%) had recurrence
Kerr-Carpenter (1990) [6]	Endoscopic bursectomy	6	6,25 (1,5-15)	Traumatic (four patients); CREST syndrome (one patient); gouty bursitis (one patient)	Olecranon + prepatellar	Two non-traumatic bursitis had relapse
Ogilvie-Harris and Gilbart (2000) [11]	Endoscopic bursectomy	50	13 (3-48)	Chronic bursitis (traumatic and rheumatoid arthritis)	Olecranon (31 patients) + prepatellar (19 patients)	Residual tenderness, pain and recurrences in prepatellar bursitis
Nussbaumer (2001) [10]	Endoscopic bursectomy	13	6	Traumatic	Olecranon (nine patients) + prepatellar (four patients)	No
Huang-Yeh (2011) [9]	Endoscopic bursectomy	60	36,3	Traumatic aseptic	Prepatellar	No
Current study	Endoscopic versus open bursectomy	49 (25/24)	16 (12-27)	Chronic bursitis (traumatic and inflamatuar arthritis)	Olecranon (30 patients) + prepatellar (19 patients)	Three open bursectomies had wound-healing problems and recurrent swelling Two endoscopic bursectomies had recurrent swelling

## Conclusions

The treatment of prepatellar and olecranon bursitis can be challenging. High complication rates and hospitalization times for the open bursectomy patients were found. Endoscopic bursectomy may be concluded to be a more reliable and accurate procedure for the treatment of prepatellar and olecranon bursitis. It offers the potentials of avoiding skin problems and scarring over the extensor surface of joints, shortens hospitalization, and minimizes anesthetic side-effects. Additionally, decreased total morbidity was found when compared to the open excision group. Besides, it is a cost-effective and more satisfying procedure. 
